# A Nomogram for Predicting the Benefit of Adjuvant Cytokine-Induced Killer Cell Immunotherapy in Patients with Hepatocellular Carcinoma

**DOI:** 10.1038/srep09202

**Published:** 2015-03-17

**Authors:** Qiu-Zhong Pan, Qi-Jing Wang, Jia-Qiang Dan, Ke Pan, Yong-Qiang Li, Yao-Jun Zhang, Jing-Jing Zhao, De-Sheng Weng, Yan Tang, Li-Xi Huang, Jia He, Shi-Ping Chen, Miao-La Ke, Min-Shan Chen, Max S. Wicha, Alfred E. Chang, Yi-Xin Zeng, Qiao Li, Jian-Chuan Xia

**Affiliations:** 1 Collaborative Innovation Center for Cancer Medicine, State Key Laboratory of Oncology in South China, Sun Yat-Sen University Cancer Center, Guangzhou, China; 2 Department of Biotherapy, Sun Yat-Sen University Cancer Center, Guangzhou, China; 3 Department of Gastrointestinal Surgery, Chengdu Fifth People's Hospital, Sichuan, China; 4 Department of Hepatobiliary Oncology, Sun Yat-sen University Cancer Center, Guangzhou, China; 5 University of Michigan Comprehensive Cancer Center, Ann Arbor, Michigan 48109, USA

## Abstract

The benefits of adjuvant cytokine-induced killer (CIK) cell immunotherapy for hepatocellular carcinoma (HCC) remain mixed among patients. Here, we constructed a prognostic nomogram to enable individualized predictions of survival benefit of adjuvant CIK cell treatment for HCC patients. Survival analysis showed that the median overall survival (OS) and progression-free survival (PFS) for patients in the hepatectomy/CIK combination group were 41 and 16 months, respectively, compared to 28 and 12 months for patients in the hepatectomy alone group (control). Based on multivariate analysis of the entire cohort, independent factors for OS were tumor size, tumor capsule, pathological grades, total bilirubin, albumin, prothrombin time, alpha-fetoprotein, and tumor number, which were incorporated into the nomogram. The survival prediction model performed well, as assessed by the c-index and calibration curve. Internal validation revealed a c-index of 0.698, which was significantly greater than the c-index value of the TNM (tumor–node–metastasis) staging systems of 0.634. The calibration curves fitted well. In conclusions, our developed nomogram resulted in more accurate individualized predictions of the survival benefit from adjuvant CIK cell treatment after hepatectomy. The model may provide valuable information to aid in the decision making regarding the application of adjuvant CIK cell immunotherapy.

Hepatocellular carcinoma (HCC), the fifth most common type of malignant tumor worldwide, is a major cause of cancer-related deaths, especially in East Asian countries^1^,^2^,^3^. Curative surgery remains the first-line therapy for HCC^4^. However, many patients are not suitable for surgery because of extensive disease or severe liver dysfunction^5^. Therefore, alternative therapeutic strategies, including radiofrequency ablation, embolization, stereotactic body radiation therapy, and chemotherapy, have been performed for HCC^2^. Unfortunately, even after these aggressive interventions, the long-term survival of HCC remains poor, due to the high incidence of intrahepatic recurrence and/or distant metastases^6^. Consequently, there is considerable interest in exploring the potential benefit of immune-based therapies for these patients.

Cytokine-induced killer (CIK) cells have demonstrated the characteristics of rapid proliferation, broad-spectrum antitumor activity, and minimal toxicity^7^,^8^ and have become a viable new treatment option in the field of cancer immunotherapy^9^,^10^,^11^. A recent series of clinical studies have been performed and showed that adjuvant CIK cell treatments can improve the overall survival (OS) and/or progression-free survival (PFS) of patients with cancer, including those with hematological malignancies or solid tumors^12^,^13^,^14^,^15^,^16^,^17^,^18^,^19^,^20^. In a prospective randomized trial by Dong et al., the PFS rate of patients who received adjuvant CIK cell treatment after hepatectomy was significantly higher than those who underwent hepatectomy alone^12^. Our recent retrospective studies also found that the addition of adjuvant CIK cell immunotherapy to surgery could improve 5-year survival probability from 50.2% for patients treated with isolated hepatectomy to 65.9% for patients treated with adjuvant CIK cell immunotherapy after surgery^19^. However, due to the rarity of large-scale multi-center prospective clinical trials and the lack of a relevant prognostic nomogram, clinicians currently often find themselves struggling in expecting whether adjuvant CIK cell immunotherapy will be beneficial to their patients. No systematic effort, to date, has been made to identify and use a combination of clinical data that could indicate which patients are likely to experience a notable survival benefit from CIK cell immunotherapy following hepatectomy while other patients should be treated only with selected modalities. Currently, the American Joint Committee on Cancer (AJCC) TNM (tumor–node–metastasis) staging system is commonly used to evaluate the prognosis of patients with HCC. However, the TNM staging system was not specifically developed for patients receiving CIK cell treatment. Consequently, establishing a novel model superior to the current TNM staging system for predicting patient survival benefits after the receipt of adjuvant CIK cell treatments is warranted.

The aims of this study were to construct a survival prediction model to be applied to predict the survival benefit of an individual HCC patient after receiving adjuvant CIK cell immunotherapy.

## Methods

### Study population

A retrospective study was performed by reviewing the medical records of patients with HCC from a computerized database at the Sun Yat-sen University Cancer Center (Guangzhou, China) between December 2001 and May 2009. The study protocol was designed in accordance with the guidelines outlined in the Declaration of Helsinki and was approved by the Ethics Committee of Sun Yat-sen University Cancer Center. Written informed consent was obtained from each patient before treatment. The preoperative diagnosis of HCC was made when at least two imaging techniques showed typical features of HCC, or one imaging technique showed positive findings together with alpha-fetoprotein (AFP) levels > 400 ng/mL, or if cytological or histological evidence was obtained. Inclusion criteria were as follows: no history of previous treatment, no history of other malignancies, adequate baseline liver and renal function, histopathologically confirmed HCC, and no clinical symptoms or signs of sepsis. Exclusion criteria included the following: a history of other malignancy, previous cancer treatment, recruitment in other clinical trial, dying within 2 months of surgery, or postoperative dysfunction in any organ. After review, 1,031 patients with HCC met these criteria and were included for further analysis. Among them, 511 cases received hepatectomy and postoperative CIK cell immunotherapy (CIK group), whereas the other 520 cases received hepatectomy alone (control group).

### Adjuvant CIK cell immunotherapy

Autologous CIK cells were prepared as we described previously^15^,^19^. After culture for 14 days, CIK cells were harvested, washed, and resuspended in 100 mL normal saline supplemented with 1% human serum albumin. Before transfer to patients, a fraction of the CIK cells were collected to evaluate the number, viability (by dye exclusion test), and possible contamination by bacteria, fungi, or endotoxins. Then, 1.0–1.5 × 10^10^ autologous CIK cells were administered to patients via intravenous infusion. Generally, patients received at least 4 cycles of CIK cell immunotherapy at an interval of 2 weeks. Patients were eligible for CIK maintenance treatment with the same procedure as described above unless the disease was in progression.

### Follow-up

After surgery, all the patients were followed-up regularly at our outpatient department. Generally, patients were observed once every 3 months for the first 2 years, every 6 months for years 3 to 5, and annually thereafter. Additionally, telephone inquiries were carried out regularly for each patient at our follow-up center. At each follow-up visit in the outpatient department, AFP and liver function tests, abdominal ultrasonography, and chest radiography were carried out. Chest computed tomography, bone scintigraphy, positron emission tomography, and biopsy were performed when tumor recurrence or metastasis were suspected. OS and PFS were used as the primary end points of interest in this study. OS was defined as the interval between surgery and death or the last known follow-up. PFS was calculated from surgery to the time of the first detectable recurrence (local or distant) or the date of the last follow-up. Treatments for recurrent tumors were determined by our multidisciplinary team that included surgeons, oncologists, radiologists, physicians, and pathologists.

### Statistical analysis

To evaluate differences between treatment groups, Student's *t*-test and the Mann–Whitney U test were used to compare continuous variables; the Pearson χ^2^ test and Fisher's exact test were used to compare categorical variables. The Kaplan–Meier method and log-rank test were used to evaluate OS and PFS, and to determine the difference. The following potential prognostic variables were assessed: age, gender, AFP, total bilirubin (TBIL), albumin (ALB), alanine aminotransferase (ALT), prothrombin time (PT), hepatitis B surface antigen (HBsAg), tumor size, tumor number, capsule, microvascular invasion, pathological grades or TNM staging. However, TNM staging was highly correlated with tumor size and tumor number, so it was excluded from the final assessment. Univariate and multivariate regression analyses were performed using Cox regression modeling, and this model formed the basis of the survival prediction model. The proportional hazards assumption of the Cox model was verified by tests of the correlation with time. Nomogram development began by identifying the most informative variables in the multivariate Cox model, which used the Akaike information criteria (AIC) as a stopping rule to arrive at a final model. The coefficients of the final model were then used to formulate a nomogram capable of predicting individual survival probability^21^.

The performance of the nomogram was assessed by measuring both discrimination and calibration. Discrimination was evaluated by calculating the c-index, which was equal to the area under the receiver operating characteristics curve for censored data^22^, with 0.5 for random prediction and 1.0 for a perfectly discriminating model. The larger the c-index, the more accurate was the prediction. Calibration, which compares the predicted survival based on the nomogram with the Kaplan–Meier estimate of survival, was evaluated using a calibration curve. Both discrimination and calibration were assessed in the entire cohort using 1000 bootstrap resamplings^23^. The predictive abilities of the nomogram and the TNM staging system were compared by computing the c-index for each method.

Statistical analyses were performed using SPSS version 16.0 (SPSS, Inc., Chicago, IL, USA) and R version 3.1.0 (http://www.r-project.org/) with library rms, Hmisc, and survival^23^. All tests were two-sided with a statistical significance level set at *p* < 0.05. The correlated computer codes for nomogram with R are listed in the supplementary materials.

## Results

### Patient demographics and clinicopathological characteristics

A total of 1,031 patients with HCC were included and divided into two cohorts (the CIK and control groups) in the final analysis. The demographic and clinicopathological characteristics were well-matched between groups (Table 1). Most patients were male (87.4%) and positive for HBsAg (84.0%); 74.5% of the patients had a single tumor nodule at the time of resection (Table 1). No statistically significant differences between the two groups were observed for age, gender, AFP, TBIL, ALB, ALT, PT, HBsAg, tumor size, tumor number, capsule, microvascular invasion, pathological grades or TNM staging(*p* > 0.05, Table 1).

### Patient prognosis

**Figure 1:**
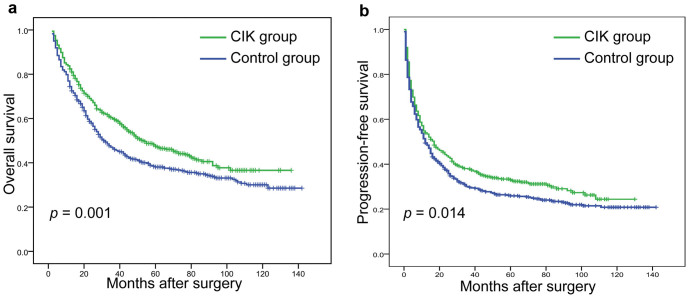
Kaplan–Meier survival curves for patients with hepatocelluar carcinoma (HCC) who received adjuvant CIK cell treatment (n = 511) or surgery alone (n = 520). (A) Actuarial overall survival (OS) grouped by cytokine-induced killer (CIK) cells. (B) Actuarial progression-free survival (PFS) grouped by CIK cells. The log-rank test showed a significantly higher OS and PFS rate in the CIK group than the control group.

Actuarial OS and PFS plots were grouped by receipt of adjuvant CIK cell immunotherapy (Fig. 1). The median OS for the entire cohort was 34 months (range, 4 to 142 months). The median OS for patients in the CIK group was 41 months compared to 28 months for patients in the control group. The 1-, 3-, and 5-year OS rates were 82.4%, 60.0%, and 47.5%, respectively, for the CIK group compared to 74.4%, 46.6%, and 38.1% for the control group (log-rank test, *p* = 0.001). The median PFS for the entire cohort was 14 months (range, 3 to 142 months). The median PFS for patients in the CIK group was 16 months compared to 12 months for patients in the control group. The 1-, 3-, and 5-year PFS rates were 54.0%, 37.8%, and 33.0%, respectively, for the CIK group compared to 49.4%, 29.9%, and 25.9% for the control group (log-rank test, *p* = 0.014).

### Prognostic nomogram for OS

**Figure 2:**
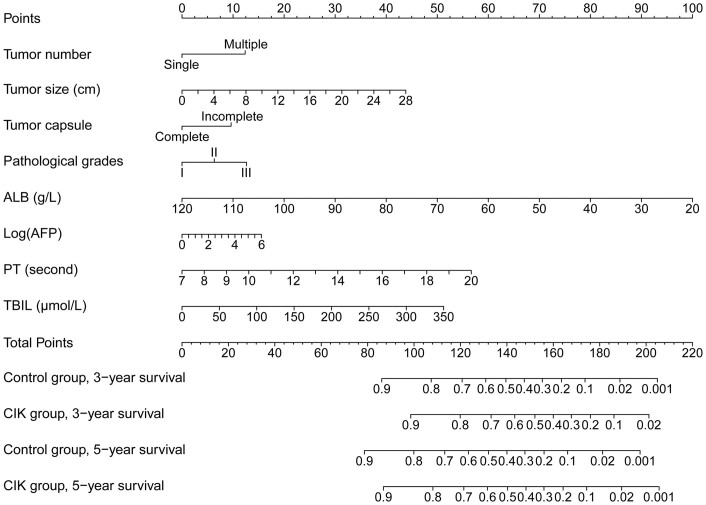
A nomogram for the prediction of 3- and 5-year overall survival for patients who receive adjuvant CIK cell treatment or surgery alone. The nomogram is used by totaling the points determined at the top of the scale for each factor. This total is then identified on the total points scale to determine the estimated probability of 3- and 5-year overall survival.

In the multivariable Cox regression models, among all 13 variables tested, 8 variables (tumor size, tumor capsule, pathological grades, AFP, ALB, PT, TBIL, and tumor number) were independent risk factors for OS and were incorporated into the nomogram to predict survival for an individual (Table 2; Fig. 2). We found that the AIC of a model with these 8 variables was lower (better) than those of any other models, so we selected this as the final model.

**Figure 3:**
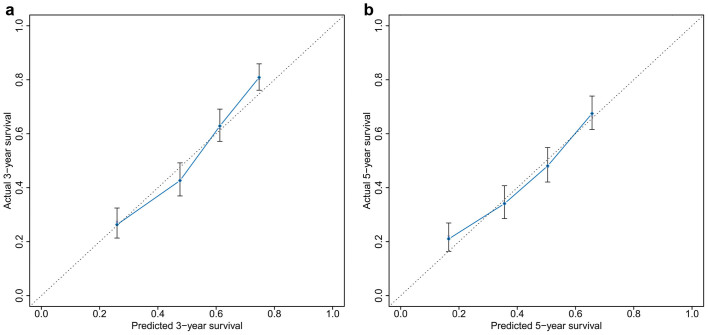
A calibration curve for predicting patient survival at (A) 3 and (B) 5 years. The calibration curve shows how the predictions from the nomogram compared to the actual outcomes for the 1,031 patients.

The performance of the nomogram was internally validated for discrimination and calibration. Discrimination, as determined by the c-index, was 0.698 (95% CI, 0.677–0.719). The calibration curve for the probability of 3- or 5- year survival showed good agreement between the prediction made by nomogram and the observed outcomes (Fig. 3).

Table 3 presents the predicted outcomes for patients with the indicated variable based on our model. For example, for a patient with a singular, II pathological grades, complete capsule tumor that is 7 cm in diameter, and a series of specific laboratory indices (their median level, i.e., ALB = 41.6 g/L, AFP = 10.9 ng/mL, PT = 13.3 second, and TBIL = 16.2 μmol/L), our model predicts that after the receipt of adjuvant CIK cell immunotherapy, the 3- and 5-year OS rate would significantly increase from 47.9% and 38.4% to 62.2% and 48.8%, respectively. By contrast, another patient with a 90 g/L ALB level and other clinical characteristics similar to the previous patient may derive some benefit from adjuvant CIK treatment, but this benefit would not be statistically significant.

### A comparison of the predictive accuracy for OS between the nomogram and TNM staging system

**Figure 4:**
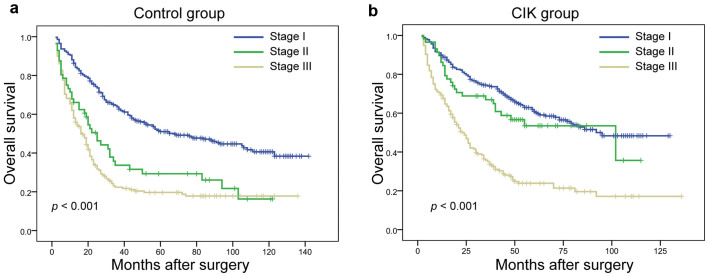
Kaplan–Meier survival curves for patients from the (A) control and (B) CIK groups, as categorized by TNM staging systems.

The TNM staging system was unsatisfactory for stratifying patients between stages II and III in the control group, and was unsatisfactory for discriminating between stages I and II in the CIK group (Fig. 4). Our nomogram showed better accuracy in predicting both short- and long-term survival in this retrospective cohort (Fig. 3). The c-index of the nomogram was 0.698, which was significantly higher (*p* < 0.05) than the c-index of the TNM staging system, which was 0.634. These results suggested that the nomogram was a useful tool for predicting the survival benefit of patients with HCC after receiving adjuvant CIK cell treatment.

## Discussion

Others and our groups have shown that the addition of adjuvant CIK cell immunotherapy to hepatectomy can reduce tumor recurrence and prolong the survival of HCC patients^12^,^19^; however, because of the lack of large, prospective, randomized controlled clinical trials, the role of adjuvant CIK cell treatment for resected HCC patients remains controversial. To date, no tool has been developed to estimate the individual survival benefit for patients with HCC who received adjuvant CIK cell treatment after hepatectomy. Thus, in this study, we developed and internally validated a nomogram using a cohort of HCC patients treated with surgery plus CIK cell treatment or surgery alone.

Currently, nomograms are increasingly being used to improve decision making^24^. Many cancer risk prediction models are being developed and used today for a variety of cancers^25^,^26^,^27^,^28^,^29^,^30^,^31^,^32^,^33^,^34^, and are useful for directing individualized therapies for a specific patient. To construct an accurate model, we collected several clinicopathological parameters related to the prognosis of HCC. After multivariate Cox regression analysis of these variables using AIC as a stopping rule, we identified eight risk factors as independent predictors for OS in HCC: tumor size, tumor capsule, pathological grades, TBIL, ALB, PT, AFP, and tumor number. Because the incorporation of multiple factors into prediction models can provide more accurate estimates^35^, all of these informative variables were used to construct a prognostic nomogram for patients with HCC. The nomogram showed a good performance for predicting the survival benefit for an individual patient with HCC after the receipt of CIK cell treatment, which was supported by the c-index (0.698) and calibration curve.

Nomograms have been reported to be more accurate than conventional staging systems for predicting prognosis in some cancers^36^,^37^. In this study, when we compared the c-index of our nomogram with the TNM staging system, we could conclude that our nomogram showed a more accurate predictive power than the TNM staging system. This finding can be explained by the fact that parameters in our model reflect some important elements in both the Child–Pugh scoring system and TNM staging system, thereby achieving better predictive accuracy.

Our study has some limitations. First, this is a retrospective study and the nomogram was constructed based on available data obtained from a single institution. Second, our nomogram needs to be externally validated using other patient databases to test its performance and reproducibility. We only validated the model internally using a bootstrap resampling method. Third, our model is only applicable to patients treated with surgery plus CIK cells or surgery alone. Whether this nomogram can be applied to patients who receive a novel treatment modality remains to be determined. Despite these limitations, our study is meaningful as it is the first nomogram developed to predict individual survival for patients with HCC who receive adjuvant CIK cell treatment followed by surgery.

We believe that our nomogram could be a useful tool for both physicians and patients for predicting the survival benefit associated with adjuvant CIK cell treatment. However, when making a final decision of whether adjuvant CIK cell treatment should be administered, the physician and patient still should engage in careful discussion because multiple factors, such as quality of life and specific patient preferences, are not taken into account in a prediction model. Moreover, with the development of personalized medicine, we believe that these types of prediction models will be increasingly important in the future as we attempt to improve outcomes by individualizing therapeutic recommendations. Thus, using our nomogram, we can estimate the survival probability for an individual patient. We will extend our study by validating this model both externally and in a prospective manner, and we will also explore the possibility of incorporating additional prognostic variables, such as the immune signature of tumor tissue, into our model to further improve its performance.

In conclusion, we have developed a nomogram that will help physicians to make an individualized estimate of the net survival benefit of adjuvant CIK cell treatment for patients with HCC. The model may aid clinicians in identifying the patients who can benefit from surgery plus CIK cell treatment compared to surgery alone.

## Author Contributions

Q.Z.P., Q.L. and J.C.X. designed the overall project. Q.J.W., J.Q.D., K.P., Y.Q.L., Y.J.Z., J.J.Z. and D.S.W. contributed the collection and assembly of data. Q.Z.P., Y.T., L.X.H., J.H., S.P.C., M.L.K., M.S.C., M.S.W., A.E.C. and Y.X.Z. contributed the data analysis and interpretation. Q.Z.P., Q.L. and J.C.X. wrote the manuscript.

## Supplementary Material

Supplementary Information Supplementary materails

## Figures and Tables

**Table 1:** Baseline characteristics of 1,031 patients, stratified according to individuals who received either surgery with CIK cell treatment (CIK group) or surgery alone (control group)

Variable	All (n = 1,031)	Control group (n = 520)	CIK group (n = 511)	*p* value
Age (years)				0.78
Median	48	48	48	
Range	13–80	15–78	13–80	
Gender				0.94
Male	901 (87.4%)	454 (87.3%)	447 (87.5%)	
Female	130 (12.6%)	66 (12.7%)	64 (12.5%)	
AFP (ng/mL)				0.96
Median	242.5	284.0	201.9	
Range	1–350,000	1–350,000	1–121,000	
TBIL (μmol/L)				0.21
Median	16.2	16.3	16.0	
Range	4.3–335.4	5.3–335.4	4.3–302.5	
ALB (g/L)				0.21
Median	41.6	41.5	41.8	
Range	24.2–114.0	27.7–52.7	24.2–114.0	
ALT (U/L)				0.87
Median	41.0	41.0	40.0	
Range	4.0–968.0	5.0–932.0	4.0–968.0	
PT (second)				0.23
Median	13.3	13.2	13.3	
Range	7.3–19.5	7.3–19.5	8.5–19.0	
HBsAg				0.58
Positive	866 (84.0%)	440 (84.6%)	426 (83.4%)	
Negative	165 (16.0%)	80 (15.4%)	85 (16.6%)	
Tumor size (cm)				0.80
Median	7.0	7.0	7.0	
Range	1.0–27.0	1.0–25.0	1.0–27.0	
Tumor number				0.17
Single	768 (74.5%)	397 (76.3%)	371 (72.6%)	
Multiple	263 (25.5%)	123 (26.7%)	140 (27.4%)	
Capsule				0.94
Incomplete	590 (57.2%)	297 (57.1%)	293 (57.3%)	
Complete	441 (42.8%)	223 (42.9%)	218 (42.7%)	
Microvascular invasion				0.37
Yes	91 (8.8%)	50 (9.6%)	41 (8.0%)	
No	940 (91.2%)	470 (90.4%)	470 (92.0%)	
Pathological grades				0.51
I	189 (18.3%)	99 (19.0%)	90 (17.6%)	
II	605 (58.7%)	296 (57.0%)	309 (60.5%)	
III	237 (23.0%)	125 (24.0%)	112 (21.9%)	
TNM staging				0.953
I	562	285	277	
II	114	56	58	
III	355	179	176	

AFP, alpha-fetoprotein; ALB, albumin; ALT, alanine aminotransferase; CIK, cytokine-induced killer cells; HBsAg, hepatitis B surface antigen; PT, prothrombin time; TBIL, total bilirubin.

**Table 2:** Univariable and multivariable Cox regression analyses for OS in 1,031 patients treated for HCC

Variables	Univariate analysis			Multivariate analysis		
	HR	95% CI	*p* value	HR	95% CI	*p* value
Age (yrs)	1.002	0.995–1.009	0.579			
Gender (male vs. female)	1.170	0.911–1.504	0.219			
TBIL (μmol/L)	1.005	1.002–1.008	<0.001	1.005	1.002–1.008	0.001
ALB (g/L)	0.947	0.928–0.966	<0.001	0.966	0.947–0.986	0.001
ALT (U/L)	1.001	0.999–1.002	0.268			
PT (second)	1.176	1.106–1.251	<0.001	1.165	1.095–1.239	<0.001
Log (AFP)	1.204	1.138–1.273	<0.001	1.096	1.033–1.163	0.002
HBsAg (positive vs. negative)	1.062	0.852–1.323	0.593			
Tumor size (cm)	1.076	1.057–1.095	<0.001	1.056	1.036–1.076	<0.001
Tumor number (multiple vs. single)	1.894	1.593–2.253	<0.001	1.540	1.286–1.844	<0.001
Tumor capsule (complete vs. incomplete)	0.589	0.507–0.706	<0.001	0.716	0.604–0.849	<0.001
Microvascular invasion (yes vs. no)	1.122	0.846–1.488	0.423			
Pathological grades						
II vs. I	1.643	1.253–2.153	<0.001	1.204	0.953–1.522	0.119
III vs. I	2.851	2.370–3.344	<0.001	1.535	1.177–2.002	0.002

AFP, alpha-fetoprotein; ALB, albumin; ALT, alanine aminotransferase; CI, confidence interval; HBsAg, hepatitis B surface antigen; HCC, hepatocellular carcinoma; HR, hazard ratio; PT, prothrombin time; TBIL, total bilirubin.

**Table 3:** Summary of Nomogram predictions

Variables	Interval	Adjuvant CIK cell treatment
Tumor size (cm)	<4.6	N.S.
	4.6–7.5	+
	>7.5	N.S.
ALB (g/L)	<38.5	N.S.
	38.5–45.0	+
	>45.0	N.S.
AFP (ng/mL)	<79.4	N.S.
	79.4–1023.3	+
	>1023.3	N.S.
PT (second)	<12.3	N.S.
	12.3–13.7	+
	>15.95	N.S.
TBIL (μmol/L)	<9.8	N.S.
	9.8–34.4	+
	>34.4	N.S.
Tumor number	Single	+
	Multiple	N.S.
Tumor capsule	Complete	N.S.
	Incomplete	+
Pathological grades	I	N.S.
	II	+
	III	N.S.

Abbreviations: AFP, alpha-fetoprotein; ALB, albumin; CIK, cytokine-induced killer cells; PT, prothrombin time; TBIL, total bilirubin; N.S., No significant net survival benefit after receiving CIK cell treatment; +, Significant net survival benefit after receiving CIK cell treatment.
