# Supplementary material for: A Nomogram for Predicting the Benefit of Adjuvant Cytokine-Induced Killer Cell Immunotherapy in Patients with Hepatocellular Carcinoma
Source: Sci Rep. 2015 Mar 17;5:9202. doi: 10.1038/srep09202 (PMC4361845; doi:10.1038/srep09202)
Supplement: Supplementary Information — Supplementary materails [file srep09202-s1.pdf]

Supplementary Information

A Nomogram for Predicting the Benefit of Adjuvant Cytokine-Induced Killer Cell Immunotherapy in Patients with Hepatocellular Carcinoma

Qiu-Zhong Pan[1, 2], Qi-Jing Wang[2], Jia-Qiang Dan[3], Ke Pan[1, 2], Yong-Qiang Li[2], Yao-Jun Zhang[4], Jing-Jing Zhao[1, 2], De-Sheng Weng[1, 2], Yan Tang[1, 2], Li-Xi Huang[2], Jia He[2], Shi-Ping Chen[2], Miao-La Ke[2], Min-Shan Chen[4], Max S. Wicha[5], Alfred E. Chang[5], Yi-Xin Zeng[1], Qiao Li[5, *], and Jian-Chuan Xia[1, 2, *]

[1] Collaborative Innovation Center for Cancer Medicine, State Key Laboratory of Oncology in South China, Sun Yat-Sen University Cancer Center, Guangzhou, China
[2] Department of biotherapy, Sun Yat-Sen University Cancer Center, Guangzhou, China
[3] Department of gastrointestinal surgery, Chengdu Fifth People's Hospital, Sichuan, China
[4] Department of hepatobiliary oncology, Sun Yat-sen University Cancer Center, Guangzhou, China
[5] University of Michigan Comprehensive Cancer Center, Ann Arbor, Michigan 48109, USA

***Correspondence to:** Jian-Chuan Xia or Qiao Li, State Key Laboratory of Oncology in South China; Department of Biotherapy, Sun Yat-sen University Cancer Center, 651 Dongfeng Road East, Guangzhou 510060, P. R. China. Tel.: +86-20-87343173, Fax: +86-20-87343392, Email: xiajch@mail.sysu.edu.cn or University of Michigan Comprehensive Cancer Center, 3520B MSRB-1, 1150 W. Medical Center Dr., Ann Arbor, MI 48109-5652, Phone: (734) 615-1977, Fax: (734) 998-2440, Email: qiaoli@umich.edu

Correlated Computer Codes for Nomogram with R

```
library(rms)

library(survival)

library(Hmisc)
```

For Cox Proportional Hazards Model

```
f<-cph(Surv(OS,Status)~ALB+PT+……+strat(CIK), surv = T, x = TRUE, y = TRUE, time.inc = 36 or 60)
```

For Nomogram

```
surv < - Survival(f)

nom<-nomogram(f, fun = list(function(x)g(36, x, stratum = 1), function(x)g(36, x, stratum = 2), function(x)g(60, x, stratum = 1), function(x)g(60, x, stratum = 2)), lp = 0,

fun.at = (c(0.001, 0.02, seq(0.1, 0.8, by = 0.1), 0.9)),

funlabel=(c("Control group, 3-year survival", "CIK group, 3-year survival", "Control group, 5-year survival", "CIK group, 5-year survival")))

par(mar = c(2,1,2,1), lty = 1, lwd = 1, font = 1)

plot(nom, xfrac = 0.35, label.every = 0.1, cex.axis = 0.8, cex.var = 0.9, lmgp = 0.2)
```

For Computing the C-Index and 95% CI

```
rcorrcens(Surv(OS,Status)~predict(f));    95%CI, 1.96 × se; se = S.D./2
```

For the Comparison of C-Index in Different Models

```
rcorrp.cens(x1, x2, S, Surv(OS, Status))
```

For Calibration Curve

```
set.seed(1)

cal<-calibrate(f, cmethod = "KM", method = "boot", u = 36 or 60, m = 257, B = 1000)

par(mar = c(8, 5, 3, 2), cex = 1.5, cex.axis = 1.5, cex.lab = 1.5)

plot(cal, lwd = 3, lty = 1, errbar.col = c(rgb(0, 0, 0, maxColorValue = 255)), xlim = c(0, 1), ylim =

c(0, 1), xlab = &, ylab = &, col = c(rgb(0, 124, 194, maxColorValue = 255)))

lines(cal[,c("mean. predicted", "KM")], type = "b", lwd = 4,

col = c(rgb(0, 124, 194, maxColorValue = 255)), pch = 23)

box(lwd = 4)

abline(0, 1, lty = 3, lwd = 4, col = c(rgb(0, 0, 0, maxColorValue = 255)))

axis(1, lwd = 4)

axis(2, lwd = 4)
```

For Resampling Validation of Nomogram

```
validate(f, method = “boot,” B = 1000, dxy = T)
```
